# Bull’s Head Sign

**DOI:** 10.31662/jmaj.2021-0129

**Published:** 2021-12-03

**Authors:** Koichiro Yamamoto, Hiroyuki Honda, Hideharu Hagiya, Fumio Otsuka

**Affiliations:** 1 Department of General Medicine, Okayama University Graduate School of Medicine, Dentistry and Pharmaceutical Sciences, Okayama, Japan

**Keywords:** anterior chest pain, SAPHO syndrome, sternoclavicular joint arthritis, ^99^Tc bone scintigraphy

A 40-year-old woman presented with anterior chest pain that had persisted for over one week. Physical assessment revealed tenderness in bilateral sternoclavicular, shoulder, and sacroiliac joints. There were no skin lesions. Serum C-reactive protein level was 1.65 mg/dL. Rheumatoid factor and anti-nuclear antibody were undetectable. Blood culture tests were negative. ^99^Tc bone scintigraphy showed “bull’s head sign”(Figure 1A), in addition to accumulation in bilateral shoulder and sacroiliac joints. Contrast-enhanced magnetic resonance imaging showed bone marrow edema in the manubrium around the right sternoclavicular joint (Figure 1B). Bone computed tomography showed hyperostosis in the bilateral first sternal cartilage (Figure 1C). Taken together, sternoclavicular osteoarthritis was suggested. We made a diagnosis of SAPHO (synovitis, acne, pustulosis, hyperostosis, and osteitis) syndrome without cutaneous lesion ^(1), (2)^. After initiating treatment with non-steroidal anti-inflammatory drugs, her symptoms gradually ameliorated.

**Figure 1.**
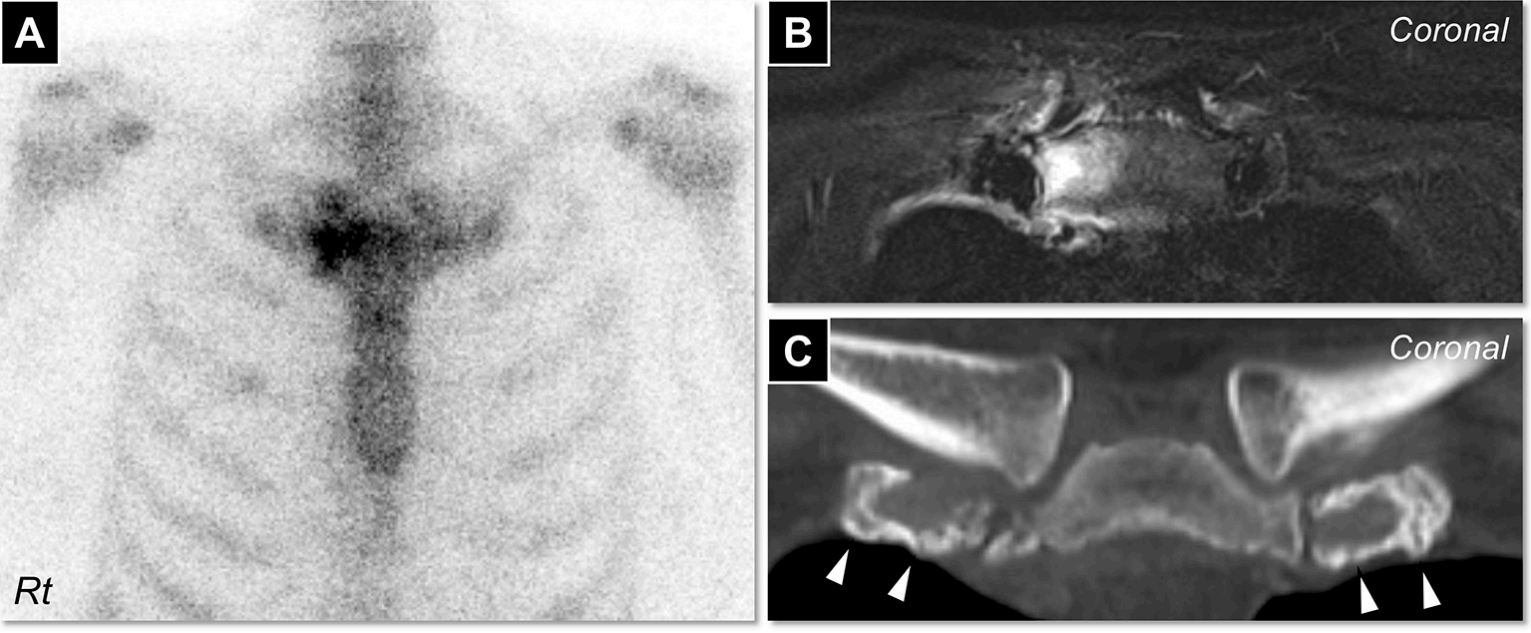
A) ^99^Tc bone scintigraphy showed “bull’s head sign.” B) Contrast-enhanced magnetic resonance imaging showed a contrast effect on the manubrium around the right sternoclavicular joint in a T2-weighted image. C) Bone computed tomography showed hyperostosis in the bilateral first sternal cartilage (arrowheads).

Osteoarticular lesions antedate skin manifestation in 32-60% of patients with SAPHO syndrome, from which at least 15% never experience cutaneous lesions ^(1), (3)^. Because “bull’s head sign” is a pathognomonic appearance of SAPHO syndrome, it should be paid attention to in patients presented with chest pain, even without dermatological manifestation ^(2), (3), (4)^.

## Article Information

### Conflicts of Interest

None

### Author Contributions

KY wrote the first draft and managed all of the submission processes. HH and HH contributed to the clinical management of the patient and revised the manuscript. FO organized the manuscript.

### Informed Consent

Written informed consent was obtained from the patient to publish this case report.
